# Causal interactions and dynamic stability between limbs while walking with imposed leg constraints

**DOI:** 10.3389/fnhum.2024.1367952

**Published:** 2024-09-05

**Authors:** Genevieve K. R. Williams, Domenico Vicinanza, Michael Attias, Stéphane Armand

**Affiliations:** ^1^Department of Public Health and Sports Sciences, University of Exeter, Exeter, United Kingdom; ^2^Faculty of Science and Engineering, Anglia Ruskin University, Cambridge, United Kingdom; ^3^School of Health Sciences, University of Applied Sciences and Arts Western Switzerland (HES-SO), Geneva, Switzerland; ^4^Kinesiology Laboratory, Geneva University Hospitals and University of Geneva, Geneva, Switzerland

**Keywords:** nonlinear dynamics, clinical gait analysis, symmetry, pathological gait, exoskeleton

## Abstract

**Aim:**

To investigate the dynamics of the motor control system during walking by examining the complexity, stability, and causal relationships of leg motions. Specifically, the study focuses on gait under both bilateral and unilateral constraints induced by a passive exoskeleton designed to replicate gastrocnemius contractures.

**Methods:**

Kinematic data was collected as 10 healthy participants walked at a self-selected speed. A new Complexity-Instability Index (CII) of the leg motions was defined as a function of the Correlation Dimension and the Largest Lyapunov Exponent. Causal interactions between the leg motions are explored using Convergent Cross Mapping.

**Results:**

Normal walking is characterized by a high mutual drive of each leg to the other, where CII is lowest for both legs (complexity of each leg motion is low and stability high). The effect of the bilateral emulated contractures is a reduced drive of each leg to the other and an increased CII for both legs. With unilateral emulated contracture, the mechanically constrained leg strongly drives the unconstrained leg, and CII was significantly higher for the constrained leg compared to normal walking.

**Conclusion:**

Redundancy in limb motions is used to support causal interactions, reducing complexity and increasing stability in our leg dynamics during walking. The role of redundancy is to allow adaptability above being able to satisfy the overall biomechanical problem; and to allow the system to interact optimally. From an applied perspective, important characteristics of functional movement patterns might be captured by these nonlinear and causal variables, as well as the biomechanical aspects typically studied.

## 1 Introduction

Walking is a fundamental human action that underpins daily activities throughout the lifespan. Examining the solutions for satisfying walking when neurological or neuromuscular impairments are present is key to understanding motor control and optimizing therapeutic strategies. Based on current understanding, the complexity and redundancy of human movement outdo our abilities to fully model its mechanics. Physical systems with time-evolving internal couplings, such as those between limb motions during gait, are commonplace in nature. However, the analytical characterisation of these motions, for example their governing equations, is only possible to determine through simplified linear models. Determining the extent to which one system drives the behavior of another through causality analysis may elegantly uncover the subtle, redundant interactions ubiquitous of biological systems that are likely to vary from stride to stride (Hausdorff et al., 1999) but are fundamental to maintaining the overall dynamic stability of the system (Orth et al., 2017; Hamacher et al., 2017; Ye et al., 2015; Wyatt et al., 2021; Strongman and Morrison, 2020).

The dynamical systems perspective contends that the timing and coordination of movements are emergent self-organized properties of the individual physical system and its interaction with the environment and the task (Newell, 1986; Kelso and Schöner, 1988). Complexity and stability are fundamental elements, characterizing a system’s dynamics with respect to attractor states (Newell, 1986; Kelso and Schöner, 1988; Kugler and Turvey, 2015; van Emmerik and van Wegen, 2000; Wade and Kazeck, 2018). Walking gait is considered a global attractor, for which complexity and stability can be estimated to capture the motor control solution (Buzzi et al., 2003; Raffalt et al., 2017; Stergiou and Decker, 2011). Based on the cyclic motion related to steps, walking can be described as a period limit cycle attractor (Buzzi et al., 2003). Complexity may be quantified by the dimensionality of a system, represented by the number of independent variables that are needed to describe its evolution (Buzzi et al., 2003; Lipsitz and Goldberger, 1992; Vicinanza et al., 2018). Specifically, a higher fractal dimension indicates a higher level of complexity, as more independent variables are needed to describe the system’s behavior. The fractal dimension can be quantified by the correlation dimension, which can be interpreted by the number of independent variables needed to describe the system’s evolution. In the study of human movement, the fractal dimension can be used to analyze and quantify the complexity of movement patterns. It can provide insights into the coordination and control of movement, as well as the adaptability and variability of human motor behavior. Meanwhile, local stability captures resilience to small perturbations, such as those naturally produced by the system during steady-state gait (Dingwell et al., 2000; Dingwell et al., 2001). Largest finite-time Lyapunov Exponent (LLE) has been used to evaluate stability in gait during steady-state walking, showing evidence that for joint angle variables, as gait speed increases local dynamic stability decreases (Dingwell et al., 2000; Dingwell and Cusumano, 2000; England and Granata, 2007), increases (Manor et al., 2008), or that an inverted U-shaped relationship exists where stability is highest at preferred stride frequency and lower at slower or faster stride frequencies (Raffalt et al., 2017; Russell and Haworth, 2014). In relation to intrinsic constraints, local dynamic stability is shown to be lower in fall-prone older adults compared to healthy older adults and young adults, even though walking speeds were slower and step lengths were smaller (Granata and Lockhart, 2008; Lockhart and Liu, 2008; Amirpourabasi et al., 2022), and decreased with age (Wade and Kazeck, 2018). The combination of complexity and stability has been theoretically linked to efficiency and adaptability in movement and is a promising area of future research (Buzzi et al., 2003; Raffalt et al., 2017; Stergiou and Decker, 2011). Therefore, in this work, we introduced an index (the Complexity and Instability Index, CII) which combines both complexity and stability as a comprehensive measure of the nonlinear dynamics characteristic related to functional biological systems that are relevant to gait stability and resilience.

Contractures can be defined by a limited extensibility or increased stiffness of the soft tissues surrounding the joints resulting in a reduced passive range of motion (ROM) (Attias et al., 2016; Fergusson et al., 2007). In order to better understand the impact of contracture on gait, experimental studies have simulated contractures on the ROM of joints of healthy participants using restrictive exoskeletons (Armand and Attias, 2019; Attias et al., 2023; Attias et al., 2019; Matjačić et al., 2006; Goodman et al., 2004; Houx et al., 2012; Harato et al., 2008; Whitehead et al., 2007; Attias et al., 2017; Attias et al., 2019). Gait adaptations have been demonstrated in Armand and Attias (Armand and Attias, 2019; Attias et al., 2023) with the aim to provide understanding of the mechanisms underpinning characteristics of pathological gait, as well as the biomechanical solutions for walking in their presence. The main results can be visualized on a web-application (pcgs.unige.ch).

This work seeks to further current understanding by exploring nonlinear causal interactions between limb motions during gait, underpinning the search for explanations or interpretations of complexities outside of mechanical relationships which might be inherently related to skill level or health status in human motor control. Therefore, the aim of this paper was to explore the causal drive and nonlinear dynamics characteristics of the legs during walking when contractures were bi- and uni- laterally emulated by a passive exoskeleton to the gastrocnemius. It is hypothesized that the unconstrained limb will predominantly drive the constrained limb, and that in constrained limbs complexity will increase and stability will decrease.

## 2 Materials and methods

### 2.1 Design of the exoskeleton

The exoskeleton, named “MIkE” (Muscle contracture Induced by an Exoskeleton), was engineered to envelop the pelvis, thigh, and shank bilaterally using custom-designed plastic cuffs and modified footwear equipped with attachment points. Incisions in the plastic cuffs were incorporated to allow direct placement of reflective markers on the skin, facilitating clinical gait analysis. The MIkE exoskeleton was designed in collaboration with the Giglio Partners Orthopedic group in Geneva to be adjustable, secure, compatible with reflective markers, non-disruptive to normal gait if no contractures are emulated, aligned with muscle action, and capable of emulating unilateral and bilateral contractures. Contractures were emulated using ropes attached to rings, to mimic the properties of natural tissues, preventing abrupt motion restrictions and ensuring a proportional limitation of the range of motion. To accommodate variations in participant size, two sizes of the exoskeleton were fabricated. The feasibility and reliability of the exoskeleton were evaluated in the precedent study by Attias et al. (2016).

### 2.2 Dataset

The data acquired during the protocol associated with Attias et al. (2016) studies (Armand and Attias, 2019; Goodman et al., 2004; Houx et al., 2012) were used for this study.

Only the data set concerning gastrocnemius contractures was used in this study. Briefly, ten healthy participants (6 females, 4 males) aged 18–35 years old (age: 27.9 ± 3.2 years; height: 1.71 ± 0.09 m; weight 64.0 ± 10.3 kg), with no known neurologic or orthopedic problems, were included in this study. Informed consent was obtained from each participant and the hospital’s institutional ethics committee approved the study protocol (CCER-13-164).

**FIGURE 1:**
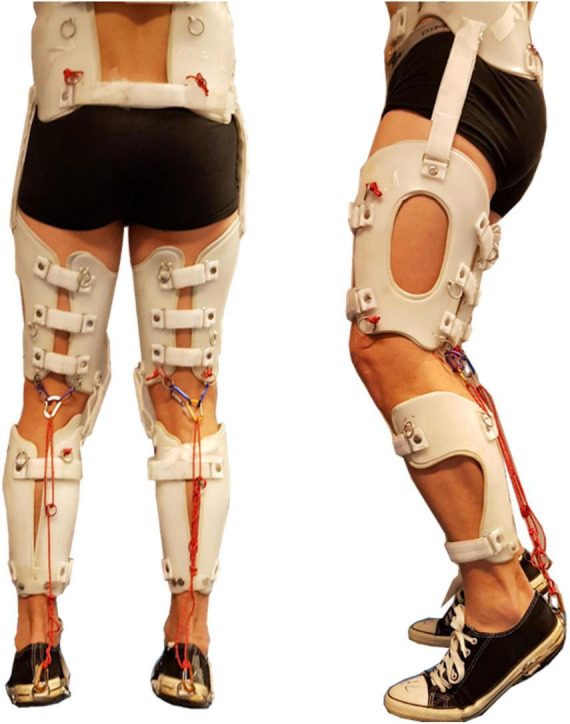
Example of device used by Attias et al. (2016) to replicate contractures on healthy participants. The exoskeleton, designed and built-in collaboration with the Giglio Partners Orthopedic group (Geneva, Switzerland) bilaterally embraced the pelvis, thigh and shank with plastic cuffs that did not occlude retroreflective markers placed on the skin. Unilateral and bilateral contractures were induced using ropes in relation to the muscle insertions and termination for the gastrocnemius muscle. In depth description of the exoskeleton design is reported in previous work (Attias et al., 2016; Goodman et al., 2004; Houx et al., 2012).

The participants were equipped with the passive exoskeleton to replicate the muscular contractures of the gastrocnemius (Figure 1). Contractures were set to 30° of plantarflexion, measured with a manual goniometer during clinical examination with the patient lying on the examination table. The three experimental conditions were walking with; no exoskeleton (normal walking), contractures on gastrocnemius on both legs (bilateral exoskeleton), and contracture on gastrocnemius on the left leg only, referred to as the constrained leg (unilateral exoskeleton). Thirty-four reflective markers were placed according to the conventional gait model (Leboeuf et al., 2019). Marker trajectories were recorded and computed with a 12-camera motion analysis system (Oqus 7+, Qualisys, Göteborg, Sweden). For each experimental condition, participants walked along a 10-meter walkway at a comfortable self-selected speed, completing four or five complete gait cycles (strides).

### 2.3 Data analysis and statistics

Positional data of knee, ankle, heel and toe markers were combined using an additive approach as described in Williams and Vicinanza (Williams and Vicinanza, 2018). State-space reconstruction of variables was created using lagged samples of the time series, according to Taken’s theorem (Takens, 2006; Huffaker et al., 2017). Time delays (T) for the reconstructions were calculated from the first minimum of the Average Mutual Information function (Fraser and Swinney, 1986). Embedding dimensions were computed from a global False Nearest Neighbors analysis (Huffaker et al., 2017).

Causality based on Convergent Cross Mapping (CCM) (Sugihara et al., 2012), was calculated to assess the driving strength of each leg to the other. In CCM, if a system Y (for example the left leg) is causally influenced by X (for example the right leg), then Y has signatures of X such that the historical measurements of Y can reliably estimate the state of X and vice versa (multispatialCCM library in R-Studio were used, with parameters: fraction of points used to calculate the asymptote of the CCM curve = 0.34, number of random libraries = 20, embedding dimension averaged 3 and time delay 19).

Complexity was assessed through correlation dimension (CD), estimated using the Grassberger and Procaccia algorithm (Grassberger and Procaccia, 1983). The correlation dimension (CD) provides a measure of complexity for the underlying phase space trajectory, where the higher the CD, the larger the number of degrees of freedom required to describe the system and the more complex.

Stability was assessed through the Largest Lyapunov Exponent (LLE) (Abarbanel et al., 1991; Rosenstein et al., 1993) calculated using the Kantz algorithm (lyap_k function from the tseriesChaos package, using 100 reference points, each with 5 neighbors within a radius of 0.6, tracked for 50 iterations, *t* = 0.5 s (Kantz and Schreiber, 2004).

A single Complexity and Instability Index (CII) was defined as the product:


CII=Correlation⁢Dimension×exp⁢(Lyapunov⁢Exponent)

CII is a mathematical combination of two key structural characteristics of nonlinear dynamical systems (CD and LLE), describing the way the system explores the state space, its dynamical stability and topological properties. To fix the ideas, in the case of an ideal pendulum CD = 1 (unidimensional attractor), LLE = 0 (perfectly stable), returning then CII = 1 x exp(0) = 1. The larger the CII, the further from ideal is the system, as a result of stronger perturbations, constraints, potentials and external forces. LLE = 0 exactly, is of course a mathematical abstraction, only attainable by a frictionless and perfectly rigid pendulum. CII = 1 is then a lower bound to CII, every real system even the most stable one, would have a CII that is *de facto* > 1, because both CD and LLE would deviate from their ideal values of 1 and 0 respectively. In our context though (walking gait), given the parameters we used for the LLE estimation (radius = 0.6 and iterations = 50 points), we are still in a domain where the LLE is close enough to 0 to consider the attractor stable.

A two-way ANOVA was performed on CII Causality with experimental condition (no exoskeleton, bilateral exoskeleton and unilateral exoskeleton) and leg (left and right, for which the left was the constrained leg and the right was the unconstrained leg in the unilateral exoskeleton condition) as the main factors, significance level set to probability (*p*) = 0.05. Tukey’s HSD *post hoc* tests were performed with a significant ANOVA statistic. Effect Size (ES) was calculated based on Cohen’s D and interpreted as small (*d* = 0.2), medium (*d* = 0.5), and large (*d* = 0.8) (Cohen, 2013). Statistics were performed in R version 4.1.2.

## 3 Results

### 3.1 Complexity and stability

Table 1 presents the CD, LLE and CII for each leg in each of the experimental conditions; no exoskeleton, bilateral exoskeleton, unilateral exoskeleton. With no exoskeleton, i.e., during normal walking, the CD of both legs was lower than the bi- and uni-lateral exoskeleton conditions. With the bilateral exoskeleton, CD is closer to two dimensional and significantly higher than with no exoskeleton for left [mean difference = 24 % higher, *p* < 0.001, ES = 1.9)] and right (mean difference = 19 % higher, *p* = 0.022, ES = 1.5). With the unilateral exoskeleton, CD remains higher than the no exoskeleton condition for the constrained leg (left leg, mean difference = 14 % higher, *p* = 0.009), but not significantly different for the unconstrained leg (right leg, *p* = 0.331).

**TABLE 1:** Mean and standard deviations of the Correlation Dimension, Largest Lyapunov exponent (LLE) and Complexity Instability Index (CII) for the left and right legs in the normal walking condition (no exoskeleton), bilateral exoskeleton and unilateral exoskeleton conditions.

	Correlation dimension		LLE		CII	
	Lleg	Rleg	Lleg	Rleg	Lleg	Rleg
	Mean (SD)	Mean (SD)	Mean (SD)	Mean (SD)	Mean (SD)	Mean (SD)[WG1]
Normal walking	1.52 (0.19)	1.50 (0.15)	0.03 (0.01)	0.04 (0.01)	1.57 (0.21)	1.55 (0.16)
Bilateral exoskeleton	1.89 (0.20)	1.78 (0.21)	0.06 (0.01)	0.06 (0.01)	2.05 (0.20)	1.88 (0.24)
Unilateral exoskeleton (left = constrained leg)	1.74 (0.16)	1.56 (0.13)	0.06 (0.01)	0.04 (0.02)	1.84 (0.18)	1.66 (0.15)

LLE is lowest (most stable) in the no exoskeleton condition (Table 1). With the bilateral exoskeleton, LLE is significantly higher (less stable) for both legs compared to no exoskeleton (for the left leg, mean difference = 100 % higher: *p* = 0.002; ES = 3; and right leg, mean difference is 50 % higher, *p* < 0.001, ES = 2). With the unilateral exoskeleton, LLE for the constrained (left) leg remains higher than the no exoskeleton condition (mean difference = 100 % higher, *p* = 0.014. ES = 3), but is not significantly different to normal walking for the unconstrained (right) leg (*p* = 0.197).

The increase in complexity and decrease in stability of each leg motion is reflected in the CII. There was a significant main effect of exoskeleton condition on CII (*F* = 5.297, *p* = 0.026), therefore post-hoc tests were performed. CII is sensitive to the effects on the nonlinear dynamical system induced by the exoskeleton conditions. Specifically, CII is significantly higher for both legs in the bilateral exoskeleton condition, compared to the no exoskeleton condition (left leg mean difference = 31 % higher, *p* < 0.001, ES = 2.3; right leg mean difference = 22 % higher, *p* < 0.001, ES = 1.6). There was no significant difference between the bilateral exoskeleton condition compared to the unilateral exoskeleton (constraint on left leg only) condition for either left (*p* = 0.229) or right leg (*p* = 0.185). In the unilateral exoskeleton condition, CII for the constrained leg was significantly lower than for the no exoskeleton condition (mean difference = 17 % lower, *p* = 0.005, ES = 1.1), however there was no significant difference between unconstrained leg in the unilateral exoskeleton condition compared to the same leg in the no exoskeleton (*p* = 0.365). The symmetry between the legs was preserved in the symmetrical conditions with no exoskeleton (*p* = 0.999) and bilateral exoskeleton (*p* = 0.438). In the unilateral exoskeleton condition, CII for the constrained leg (left) was higher but not significantly higher than for the unconstrained leg (right, *p* = 0.372).

### 3.2 Causality

Causality refers to the relationship between cause and effect, where one event or variable influences another, leaving a footprint in the dynamics of the other. In the field of human movement science, understanding causality is crucial for studying the factors that contribute to movement patterns and behaviors. One method used to investigate causality is convergent cross mapping (CCM), a statistical test that aims to identify cause-and-effect relationships between two variables that utilizes the concept of time delay embedding. The method involves constructing a time delay embedding from the time series of one variable and estimating the values of another variable from this embedding. The ability to accurately estimate the values of the second variable from the embedding quantifies how much information about the second variable has been encoded into the first variable. This quantification helps determine the causal effect of the first variable on the second variable.

In the normal walking condition, there was the strongest mutual drive of each leg to the other, suggesting that the leg-leg system is able to influence itself bilaterally and a strong coupling exists. With the bilateral exoskeleton, the drive of each leg to the other reduces (significantly compared to the no exoskeleton condition, *p* = 0.003 left to right, and *p* < 0.001 right to left), indicating a mutual decrease in the legs ability to drive each other. In the unilateral exoskeleton condition, the constrained leg (left) strongly drives the unconstrained leg, but the drive of the unconstrained leg to the constrained leg is reduced compared to no exoskeleton condition (*p* < 0.001).

## 4 Discussion

**FIGURE 2:**
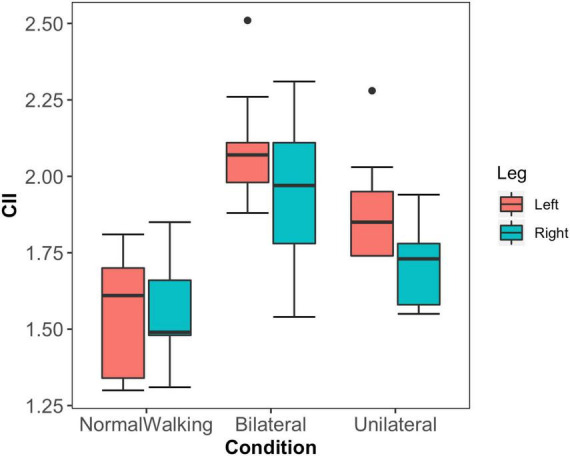
Mean and standard deviations of the Complexity Instability Index (CII) for the left (red) and right (green) leg in the three experimental conditions; the normal walking condition (No_Exoskeleton), bilateral exoskeleton (Bilateral) and unilateral exoskeleton (Unilateral; where the left leg is the constrained leg) condition. In the unilateral exoskeleton condition, the left leg is the constrained leg.

The aim of this paper was to explore the causal drive and nonlinear dynamic characteristics of the legs during walking when contractures were bi- and uni- laterally emulated by a passive exoskeleton to the gastrocnemius. During normal walking, there is a high mutual drive from one leg to the other. The CII is low, where the complexity of each leg motion is low and the stability is high (Figure 2 and Table 1). In line with the first part of the hypothesis, in bilateral exoskeleton condition, the causal drive of each leg to the other was reduced, and the CII for both legs was significantly increased. Contrary to the hypothesis, in the unilateral exoskeleton condition, the mechanically constrained leg strongly drives the unconstrained leg, and CII was significantly higher for the constrained leg compared to normal walking.

Fundamental mechanical links exist in inter-limb relations during walking, as well as subtle, redundant interactions ubiquitous of biological systems that are likely to vary from stride to stride (Hausdorff et al., 1999) but are essential to maintaining the overall dynamic stability of the system. The analysis of causality in inter-limb motion transcends information gained from pure mechanics, mean phase relations or simply unpacking the dynamics of individual limbs or segments signals. Rather, in biological systems, identifying the nature of nonlinear causal interactions between limb motions outside of mechanical relationships is likely inherently related to health status in motor control, and underpins the search for explanations or interpretations of complexities. When applied in this special case, we gain understanding of the solution of our coupled leg-leg system in satisfying walking gait in constrained conditions that reflect common pathological states, capturing how the causal relations and nonlinear dynamics are altered with the exoskeleton. Complexity and stability in actions are the hallmarks of dynamics, where complexity is represented by the number of independent variables that are needed to describe the system’s evolution in time (Vicinanza et al., 2018) and local stability as a measure of the strength of an attractor state (Dingwell et al., 2001; Dingwell and Cusumano, 2000). By considering the effects of induced contractures on the complexity and stability of leg actions during walking, we can have a window into understanding the effect of the contractures on the dynamics of this system.

**FIGURE 3:**
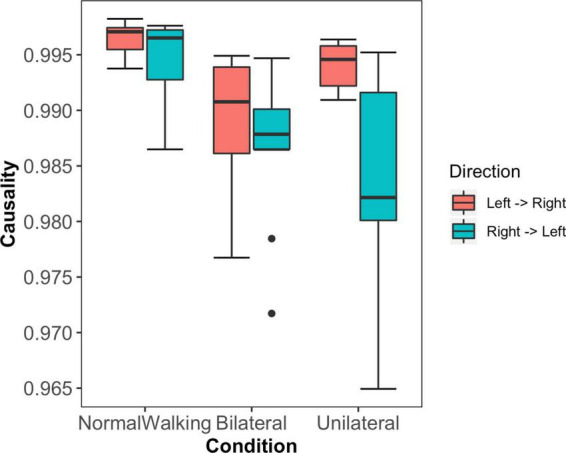
Mean and standard deviations of the causal drive of the left to the right leg (red) and right to left leg (green) across the three experimental conditions; the normal walking condition (NormalWalking), bilateral exoskeleton (Bilateral) and unilateral exoskeleton (Unilateral; where the left leg is the constrained leg) conditions.

During normal walking (no exoskeleton condition), there is a high causal drive of the action of each leg to the other, where each leg has a low CII (Figure 3 and Table 1). The effect of the bilateral exoskeleton is a reduction in strength of the causal drive of each leg to the other, and an increase in CII. These results strongly suggest that our biological system in its normal state uses redundancy to support causal interactions, which reduces complexity and increases stability in our leg dynamics during walking. In a broader sense, there might be initial evidence that the biological system in its normal redundant state uses causal interactions to reduce complexity and increase stability in our leg dynamics during walking.

The goal (natural) state of the systems complexity and stability demonstrates evidence of basic dynamical principles applied to human motor control. It is observed in tasks such as finger wagging, arm and leg swinging, and even during interpersonal coordination, that strict natural, simple and stable coupling relationships exist (Haken et al., 1985; Kelso et al., 1986). Causal aspects calculated here demonstrate motor control includes strong causal links for limbs for which CII remains low (Figure 3 and Table 1), and these characteristics are supported by redundancy. It seems then that the role of redundancy is to allow adaptability above being able to satisfy the overall biomechanical problem; and to allow the system to interact optimally. Redundancy allows the system to be influenced and driven by an externally or internally constrained subsystem in a degenerate manner. This is an important theoretical and applied point because it captures the notions of flexibility within biology being key to success, and highlights that in the instance of injury or disability the most important characteristics of *functional* motion might be captured by these nonlinear and causal variables, as well as the biomechanical aspects typically studied.

Causal drive between legs in the bilateral exoskeleton condition remains mutually low while the CII increases for both legs compared to normal walking (Figure 3 and Table 1). However, there is an asymmetrical increase where the left leg has higher CII than the right leg. Since both legs are constrained equally, this finding might reflect natural asymmetry associated with leggedness which is emphasized in bilaterally constrained conditions. Some evidence of natural asymmetry increasing with walking speed exists (Plotnik et al., 2013), however since most studies of gait symmetry have examined normal walking versus natural or imposed bilateral asymmetry (Kwek and Williams, 2021; Shorter et al., 2008) these findings require further investigation.

In comparison, when walking with the unilateral exoskeleton, the mechanically constrained leg (in this case the left leg) strongly drives the unconstrained leg (right leg), while the drive of the unconstrained leg to the constrained leg is limited (Figure 3). This nontrivial finding suggests when one leg is constrained, the system exploits redundancy in unconstrained parts of the system to be used in a compensatory rather than a driving capacity. In this scenario, we observe that exploiting redundancy facilitates an alternative technique that can be used to compensate for the constrained leg. From a theoretical standpoint, the motor control solution is then defined in light of the constraints imposed, and adaptability and exploiting redundancy are key to supporting functionality. Based on the large effect sizes reported, we provide strong evidence of this phenomenon in the current context, where constraints are imposed on healthy individuals. This finding is significant in a practical sense since optimal walking gait may be driven by the most constrained leg, and while less resembling ‘healthy’ walking kinematics, may provide a more optimal motor control solution. Indeed, some patients with unilateral paretic limb have greater deviation from normal, or greater gait impairment, on the non-paretic limb, which is hypothesized to be due to compensation strategies employed to accommodate for reduced function in the paretic limb (Devetak et al., 2016; Jarvis et al., 2022). The nonlinear aspects of leg motion or inter-leg interaction are not well studied, but could provide interesting insights into the underpinnings of the strategy.

Furthermore, it may be conjectured that the more severe the constraint, the stronger the unidirectional causal drive. While the causal drive from the constrained leg to the unconstrained leg is strong, we have evidence of a non-optimal solution since the CII for both legs remains high; which could be interpreted as the price for the compensatory nature of the actions (Figure 2). CII provided further evidence of an increase in complexity and a decrease in stability being associated with a non-optimal state of the human system during movement (Hausdorff et al., 1999; Buzzi et al., 2003; Stergiou and Decker, 2011; Vicinanza et al., 2018; Dingwell et al., 2001). Specifically, during normal walking, CII is low where the complexity of each leg motion is low, and the stability is high (Table 1). Theoretically, this pattern then allows both efficiency and adaptability and is in line with the findings of Raffalt et al. (2017), who showed minimum values of CD and LLE of kinematic variables when walking at preferred speeds. With bilateral exoskeleton conditions, a significantly higher CII for both legs was recorded, while when walking with the unilateral exoskeleton conditions, CII was significantly higher for the constrained leg compared to normal walking. This suggests that the redundancy available is used to stabilize the system components but that the compensatory actions evidenced by the causality analysis leave the system with higher CII characteristics.

Detecting causal interactions in complex biological systems is essential for understanding how these systems function and will be a promising area to understand human motor control. However, it is unknown whether a longer period of adaptation would change walking behavior in the exoskeleton conditions.

This study has several limitations, data were collected for a number of trials overground, rather than continuously. There is also a limited number of participants in the study.

## 5 Conclusion

Providing a new perspective on the theoretical notion of flexibility within biology, this work provides evidence that our biological system uses redundancy to support causal interactions, reducing complexity and increasing stability in our leg dynamics during walking. Normal walking was characterized by a high mutual drive of each leg to the other, where CII was lowest for both legs (complexity of each leg motion estimated with CD was low, and the stability estimated with LLE was high). The effect of the bilateral emulated contractures was a reduced drive of each leg to the other and an increased CII for both legs. With unilateral emulated contracture, the mechanically constrained leg strongly drives the unconstrained leg, and CII was significantly higher for the constrained leg compared to normal walking. From a dynamics system perspective, it is seen here that a constrained limb drives the free parts of the system, a nontrivial finding, suggesting that the role of redundancy is to allow adaptability above being able to satisfy the overall biomechanical problem; to allow the system to interact optimally.

From an applied perspective, in the instance of imposed constraint (injury or disability), the most important characteristics of functional motor control and the resulting motions might be captured by these nonlinear and causal variables, as well as the biomechanical aspects typically studied. Through understanding of nonlinear causal interactions between limb motions, we can drive the search for explanations or interpretations of complexities outside of mechanical relationships which might be inherently related to skill level, functionality or health status in human motor control.

## Data Availability

The raw data supporting the conclusions of this article will be made available by the authors, without undue reservation.

## References

[B1] Abarbanel H. Brown R. Kennel M. (1991). Lyapunov exponents in chaotic systems: Their importance and their evaluation using observed data. *Int. J. Modern Phys. B* 5 1347–1375. 10.1142/S021797929100064X

[B2] Amirpourabasi A. Lamb S. Chow J. Williams G. (2022). Nonlinear dynamic measures of walking in healthy older adults: A systematic scoping review. *Sensors* 22:4408. 10.3390/s22124408 PMC9228430 35746188

[B3] Armand S. Attias M. (2019). *Contracture and gait deviations.* Cham: Springer, 10.1007/978-3-319-30808-1_200-1

[B4] Attias M. Bonnefoy-Mazure A. De Coulon G. Cheze L. Armand S. (2016). Feasibility and reliability of using an exoskeleton to emulate muscle contractures during walking. *Gait Post.* 50 239–245. 10.1016/j.gaitpost.2016.09.016 27665088

[B5] Attias M. Bonnefoy-Mazure A. De Coulon G. Cheze L. Armand S. (2023). Toe-walking and its impact on first and second rocker in gait patterns with different degrees of artificially emulated soleus and gastrocnemius contracture. *Gait Post.* 105 104–109. 10.1016/j.gaitpost.2023.07.285 37523808

[B6] Attias M. Bonnefoy-Mazure A. De Coulon G. Cheze L. Armand S. (2019). Kinematics can help to discriminate the implication of iliopsoas, hamstring and gastrocnemius contractures to a knee flexion gait pattern. *Gait Post.* 68 415–422. 10.1016/j.gaitpost.2018.12.029 30594869

[B7] Attias M. Bonnefoy-Mazure A. De Coulon G. Cheze L. Armand S. (2017). Influence of different degrees of bilateral emulated contractures at the triceps surae on gait kinematics: The difference between gastrocnemius and soleus. *Gait Post.* 58 176–182. 10.1016/j.gaitpost.2017.07.118 28797961

[B8] Buzzi U. Stergiou N. Kurz M. Hageman P. Heidel J. (2003). Nonlinear dynamics indicates aging affects variability during gait. *Clin. Biomech.* 18 435–443. 10.1016/S0268-0033(03)00029-9 12763440

[B9] Cohen J. (2013). *Statistical power analysis for the behavioral sciences.* Milton Park: Routledge.

[B10] Devetak G. Martello S. de Almeida J. Correa K. Iucksch D. Manffra E. (2016). Reliability and minimum detectable change of the gait profile score for post-stroke patients. *Gait Post.* 49 382–387. 10.1016/j.gaitpost.2016.07.149 27497756

[B11] Dingwell J. Cusumano J. (2000). Nonlinear time series analysis of normal and pathological human walking. *Chaos* 10 848–863. 10.1016/S0021-9290(00)00092-0 12779434

[B12] Dingwell J. Cusumano J. Cavanagh P. Sternad D. (2001). Local dynamic stability versus kinematic variability of continuous overground and treadmill walking. *J. Biomech. Eng.* 123 27–32. 10.1115/1.1336798 11277298

[B13] Dingwell J. Cusumano J. Sternad D. Cavanagh P. (2000). Slower speeds in patients with diabetic neuropathy lead to improved local dynamic stability of continuous overground walking. *J. Biomech.* 33 1269–1277. 10.1016/S0021-9290(00)00092-0 10899337

[B14] England S. Granata K. (2007). The influence of gait speed on local dynamic stability of walking. *Gait Post.* 25 172–178. 10.1016/j.gaitpost.2006.03.003 16621565 PMC1785331

[B15] Fergusson D. Hutton B. Drodge A. (2007). The epidemiology of major joint contractures: A systematic review of the literature. *Clin. Orthop. Relat. Res.* 456 22–29. 10.1097/BLO.0b013e3180308456 17179779

[B16] Fraser A. Swinney H. (1986). Independent coordinates for strange attractors from mutual information. *Phys. Rev. A* 33:1134. 10.1103/PhysRevA.33.1134 9896728

[B17] Goodman M. Menown J. West J. M. Barr K. M. Vander Linden D. W. McMulkin M. L. (2004). Secondary gait compensations in individuals without neuromuscular involvement following a unilateral imposed equinus constraint. *Gait Post.* 20 238–244. 10.1016/j.gaitpost.2003.09.005 15531170

[B18] Granata K. Lockhart T. (2008). Dynamic stability differences in fall-prone and healthy adults. *J. Electromyogr. Kinesiol.* 18 172–178. 10.1016/j.jelekin.2007.06.008 17686633 PMC2895268

[B19] Grassberger P. Procaccia I. (1983). Characterization of strange attractors. *Phys. Rev. Lett.* 50:346. 10.1103/PhysRevLett.50.346

[B20] Haken H. Kelso J. Bunz H. (1985). A theoretical model of phase transitions in human hand movements. *Biol. Cybern.* 51 347–356. 10.1007/BF00336922 3978150

[B21] Hamacher D. Hamacher D. Müller R. Schega L. Zech A. (2017). Exploring phase dependent functional gait variability. *Hum. Mov. Sci.* 52 191–196. 10.1016/j.humov.2017.02.006 28237654

[B22] Harato K. Nagura T. Matsumoto H. Otani T. Toyama Y. Suda Y. (2008). Knee flexion contracture will lead to mechanical overload in both limbs: A simulation study using gait analysis. *Knee* 15 467–472. 10.1016/j.knee.2008.07.003 18760608

[B23] Hausdorff J. Zemany L. Peng C. Goldberger A. (1999). Maturation of gait dynamics: Stride-to-stride variability and its temporal organization in children. *J. Appl. Physiol.* 86 1040–1047. 10.1152/jappl.1999.86.3.1040 10066721

[B24] Houx L. Brochard S. Lempereur M. Remy-Neris O. (2012). Simulation of unilateral equinus using an adjustable orthosis in children: Design, feasibility and biomechanical effects. *Prosth. Orth. Int.* 36 131–136. 10.1177/0309364611427764 22080593

[B25] Huffaker R. Huffaker R. Bittelli M. Rosa R. (2017). *Nonlinear time series analysis with R.* Oxford: Oxford University Press.

[B26] Jarvis H. Brown S. Butterworth C. Jackson K. Clayton A. Walker L. (2022). The gait profile score characterises walking performance impairments in young stroke survivors. *Gait Post.* 91 229–234. 10.1016/j.gaitpost.2021.10.037 34741933

[B27] Kantz H. Schreiber T. (2004). *Nonlinear time series analysis.* Cambridge: Cambridge university press.

[B28] Kelso J. Schöner G. (1988). Self-organization of coordinative movement patterns. *Hum. Mov. Sci.* 7 27–46. 10.1016/0167-9457(88)90003-6

[B29] Kelso J. Scholz J. Schöner G. (1986). Nonequilibrium phase transitions in coordinated biological motion: Critical fluctuations. *Phys. Lett. A* 118 279–284. 10.1016/0375-9601(86)90359-2

[B30] Kugler P. Turvey M. (2015). *Information, natural law, and the self-assembly of rhythmic movement.* Milton Park: Routledge.

[B31] Kwek J. Williams G. (2021). Age-based comparison of gait asymmetry using unilateral ankle weights. *Gait Post.* 87 11–18. 10.1016/j.gaitpost.2021.01.018 33872954

[B32] Leboeuf F. Baker R. Barré A. Reay J. Jones R. Sangeux M. (2019). The conventional gait model, an open-source implementation that reproduces the past but prepares for the future. *Gait Post.* 69 235–241. 10.1016/j.gaitpost.2019.04.015 31027876

[B33] Lipsitz L. Goldberger A. (1992). Loss of complexity and aging: Potential applications of fractals and chaos theory to senescence. *JAMA* 267 1806–1809. 10.1001/jama.1992.03480130122036 1482430

[B34] Lockhart T. Liu J. (2008). Differentiating fall-prone and healthy adults using local dynamic stability. *Ergonomics* 51 1860–1872. 10.1080/00140130802567079 19034782 PMC2892176

[B35] Manor B. Wolenski P. Li L. (2008). Faster walking speeds increase local instability among people with peripheral neuropathy. *J. Biomech.* 41 2787–2792. 10.1016/j.jbiomech.2008.07.006 18706561

[B36] Matjačić Z. Olenšek A. Bajd T. (2006). Biomechanical characterization and clinical implications of artificially induced toe-walking: Differences between pure soleus, pure gastrocnemius and combination of soleus and gastrocnemius contractures. *J. Biomech.* 39 255–266. 10.1016/j.jbiomech.2004.11.024 16321627

[B37] Newell K. M. (1986). “Constraints on the development of coordination,” in *Motor development in children: Aspects of coordination and control*, eds Wade M. G. Whiting H. T. A. (Dordrecht: Martinus Nijhoff), 341–360.

[B38] Orth D. Van der Kamp J. Memmert D. Savelsbergh G. (2017). Creative motor actions as emerging from movement variability. *Front. Psychol.* 8:1903. 10.3389/fpsyg.2017.01903 29163284 PMC5671646

[B39] Plotnik M. Bartsch R. Zeev A. Giladi N. Hausdorff J. (2013). Effects of walking speed on asymmetry and bilateral coordination of gait. *Gait Post.* 38 864–869. 10.1016/j.gaitpost.2013.04.011 23680424 PMC4047486

[B40] Raffalt P. Guul M. Nielsen A. Puthusserypady S. Alkjaer T. (2017). Economy, movement dynamics, and muscle activity of human walking at different speeds. *Sci. Rep.* 7 1–4. 10.1038/srep43986 28272484 PMC5341064

[B41] Rosenstein M. Collins J. De Luca C. J. (1993). A practical method for calculating largest Lyapunov exponents from small data sets. *Phys. D* 65 117–134. 10.1016/0167-2789(93)90009-P

[B42] Russell D. Haworth J. (2014). Walking at the preferred stride frequency maximizes local dynamic stability of knee motion. *J. Biomech.* 47 102–108. 10.1016/j.jbiomech.2013.10.012 24210850

[B43] Shorter K. Polk J. Rosengren K. Hsiao-Wecksler E. T. (2008). A new approach to detecting asymmetries in gait. *Clin. Biomech.* 23 459–467. 10.1016/j.clinbiomech.2007.11.009 18242805

[B44] Stergiou N. Decker L. (2011). Human movement variability, nonlinear dynamics, and pathology: Is there a connection? *Hum. Mov. Sci.* 30 869–888. 10.1016/j.humov.2011.06.002 21802756 PMC3183280

[B45] Strongman C. Morrison A. A. (2020). scoping review of non-linear analysis approaches measuring variability in gait due to lower body injury or dysfunction. *Hum. Mov. Sci.* 69:102562. 10.1016/j.humov.2019.102562 31989953

[B46] Sugihara G. May R. Ye H. Hsieh C. Deyle E. Fogarty M. (2012). Detecting causality in complex ecosystems. *Science* 338 496–500. 10.1126/science.1227079 22997134

[B47] Takens F. (2006). *Detecting strange attractors in turbulence: Dynamical systems and turbulence, Warwick 1980 1981.* Berlin: Springer, 366–381.

[B48] van Emmerik R. van Wegen E. (2000). On variability and stability in human movement. *J. Appl. Biomech.* 16 394–406. 10.1123/jab.16.4.394

[B49] Vicinanza D. Newell K. Irwin G. Smith L. Williams G. (2018). Limit cycle dynamics of the gymnastics long swing. *Hum. Mov. Sci.* 57 217–226. 10.1016/j.humov.2017.12.014 29291544

[B50] Wade M. Kazeck M. (2018). Developmental coordination disorder and its cause: The road less travelled. *Hum. Mov. Sci.* 57 489–500. 10.1016/j.humov.2016.08.004 27876401

[B51] Whitehead C. Hillman S. Richardson A. Hazlewood M. Robb J. (2007). The effect of simulated hamstring shortening on gait in normal subjects. *Gait Post.* 26 90–96. 10.1016/j.gaitpost.2006.07.011 16949826

[B52] Williams G. Vicinanza D. (2018). Coordination in gait: Demonstration of a spectral approach. *J. Sports Sci.* 36 1768–1775. 10.1080/02640414.2017.1416974 29243945

[B53] Wyatt H. Vicinanza D. Newell K. Irwin G. Williams G. (2021). Bidirectional causal control in the dynamics of handstand balance. *Sci. Rep.* 11 1–9. 10.1038/s41598-020-79730-z 33432011 PMC7801474

[B54] Ye H. Deyle E. Gilarranz L. Sugihara G. (2015). Distinguishing time-delayed causal interactions using convergent cross mapping. *Sci. Rep.* 5 1–9. 10.1038/srep14750 26435402 PMC4592974

